# Cellulose‐Based Radiative Cooling and Solar Heating Powers Ionic Thermoelectrics

**DOI:** 10.1002/advs.202206510

**Published:** 2023-01-16

**Authors:** Mingna Liao, Debashree Banerjee, Tomas Hallberg, Christina Åkerlind, Md Mehebub Alam, Qilun Zhang, Hans Kariis, Dan Zhao, Magnus P. Jonsson

**Affiliations:** ^1^ Laboratory of Organic Electronics Department of Science and Technology Linköping University Norrköping SE‐601 74 Sweden; ^2^ Wallenberg Wood Science Center Linköping University Norrköping SE‐601 74 Sweden; ^3^ Department of Electro‐Optical systems FOI‐Swedish Defense Research Agency Linköping SE‐583 30 Sweden

**Keywords:** cellulose, ionic thermoelectrics, IR emissivity controlling, radiative cooling, solar heating

## Abstract

Cellulose opens for sustainable materials suitable for radiative cooling thanks to inherent high thermal emissivity combined with low solar absorptance. When desired, solar absorptance can be introduced by additives such as carbon black. However, such materials still shows high thermal emissivity and therefore performs radiative cooling that counteracts the heating process if exposed to the sky. Here, this is addressed by a cellulose‐carbon black composite with low mid‐infrared (MIR) emissivity and corresponding suppressed radiative cooling thanks to a transparent IR‐reflecting indium tin oxide coating. The resulting solar heater provides opposite optical properties in both the solar and thermal ranges compared to the cooler material in the form of solar‐reflecting electrospun cellulose. Owing to these differences, exposing the two materials to the sky generated spontaneous temperature differences, as used to power an ionic thermoelectric device in both daytime and nighttime. The study characterizes these effects in detail using solar and sky simulators and through outdoor measurements. Using the concept to power ionic thermoelectric devices shows thermovoltages of >60 mV and 10 °C temperature differences already at moderate solar irradiance of ≈400 W m^−2^.

## Graphical abstract

A lateral ionic thermoelectric device is driven by radiative cooling and solar heating using cellulose‐based cooler and heater materials. The concept produces temperature differences and thermovoltages both during daytime and nighttime, with the latter owing to suppression of thermal emission from the heater using a visibly transparent MIR‐reflecting coating.

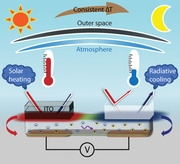

## 1 Introduction

Cellulose is the most abundant natural organic material on earth. It is a valuable renewable and sustainable resource accompanied by many beneficial properties such as biocompatibility, biodegradability, nontoxicity, and thermal and chemical stability.^[^ ^1^, ^2^ ^]^ In addition to these intrinsic merits, cellulose is a versatile biopolymer and has gained attention for many applications due to its variable properties tuned by suitable solvent systems and processes. Not least, many studies demonstrated different interactions of light with cellulose and its derivatives for various applications such as light guidance, sensors, optoelectronics, and solar‐regulating building technologies.^[^ ^3^, ^4^, ^5^, ^6^, ^7^ ^]^ One of the most interesting optical properties of cellulose is its inherent non‐absorptance to visible light. This opens for non‐absorptive cellulose films that are designed to be either transparent,^[^ ^8^ ^]^ hazy,^[^ ^9^ ^]^ reflective,^[^ ^10^ ^]^ or even colored,^[^ ^11^ ^]^ by varying its structure at the micro‐ and nanoscales, or by the addition of scatterers.^[^ ^12^, ^13^ ^]^ Solar absorption and solar‐induced heating can also be introduced in a controllable manner by the addition of absorptive components such as carbon quantum dots^[^ ^14^ ^]^ or carbon nanotubes.^[^ ^15^ ^]^

By contrast to its visible non‐absorptive behavior, molecular stretching vibrations make cellulose absorptive in the mid‐infrared (MIR) spectral range. Because absorptance equals emissivity according to Kirchhoff's law of thermal radiation, this makes cellulose suitable as thermal emitter in the range ≈10 µm at which objects at room temperature emit Planck radiation. This range also coincides with a transparency window of the atmosphere (8‐14 µm). Together, these aspects form the basis for the concept of radiative cooling, by which objects on earth are cooled by emitting thermal radiation through the atmosphere and to the cold outer space. There is no need for any external energy input for this sustainable cooling method, which highlights its potential to reduce the global energy consumption.^[^ ^16^ ^]^ Several studies have recently utilized the strong thermal emissivity of cellulose for radiative cooling applications, including reflective systems that also suppress solar‐induced heating to achieve subambient cooling during daytime. As example, continuous sub‐ambient cooling during both day and night was demonstrated based on delignified and densified wood, which backscattered solar radiation while emitting strongly in the MIR range.^[^ ^17^ ^]^ Likewise, Juliana et al. synthesized cellulose acetate porous scattering films with low absorption of solar irradiance and strong MIR emittance. Field tests demonstrated films reaching up to ≈5 °C below ambient temperature and it was estimated that the films can reach average minimum temperatures of 7–8 °C below the ambient.^[^ ^18^ ^]^ Our previous work reported similar cellulose‐based reflective porous coolers and compared them with transparent homogenous nanocellulose coolers.^[^ ^19^ ^]^ While reflective coolers are suitable to cool objects and simultaneously protect them from solar radiation, such transparent coolers are instead designed as coatings for optoelectronic devices like solar cells. Addition of visible scatters to homogenous nanocellulose films further enabled hazy transparent coolers,^[^ ^19^ ^]^ and we demonstrated that self‐assembled cellulose nanocrystal films can provide structurally colored trans‐reflective coolers.^[^ ^20^ ^]^

Besides thermal regulation, recent research explored ways to harvest energy from (non‐cellulose based) radiative cooling through the thermoelectric effect.^[^ ^21^, ^22^, ^23^, ^24^ ^]^ This complements the large body of research on powering thermoelectric (and pyroelectric) devices by solar heating of cellulose‐based and non‐cellulose materials.^[^ ^25^, ^26^, ^27^ ^]^ Solar heating and radiative cooling were also recently combined to generate temperature differences in thermoelectric generators during both daytime and nighttime.^[^ ^28^, ^29^ ^]^ However, previous research was limited to combining radiative cooling with commercial thermoelectrics based on traditional inorganic materials, which poses limitations of cost, performance and sustainability. In turn, research on ionic thermoelectrics has experienced rapid growth recently owing to not only environmentally friendly material compositions but also superior Seebeck coefficients and correspondingly much larger thermovoltages than provided by electronic thermoelectric systems.^[^ ^30^, ^31^, ^32^ ^]^ Here, we demonstrate the first ionic thermoelectric device driven by solar heating and radiative cooling. Both the cooler and heater are based on cellulose, purposefully designed to have contrasting optical properties in both the visible and thermal spectral ranges. As cooler material, we use cellulose acetate films fabricated by electrospinning, which provides not only high thermal emissivity but also efficient broadband reflectance throughout the visible region due to a highly porous structure. This makes the material effective both for radiative cooling and in suppressing heat generation from solar absorption. As heater material, we instead designed homogenous cellulose nanofiber films containing carbon black, to minimize scattering of solar light and maximize solar absorption and heat generation. Moreover, we designed the heater material to minimize its radiative cooling using an indium tin oxide (ITO) top layer, which is visibly transparent to allow solar absorption but reflects MIR light and thereby reduces the thermal emissivity of the material. We optimize and characterize both heater and cooler materials in terms of optical properties and by monitoring their temperatures when exposed to the sun, the sky, or both. In that respect, we use both solar and sky simulators for detailed characterization, and employ outdoor measurements for practical demonstrations. Thanks to the opposite optical and thermal behavior of the cooler and heater, they enabled the construction of cellulose‐based ionic thermoelectric devices that generate temperature gradients and thermovoltages both during daytime and nighttime.

**Figure 1:**
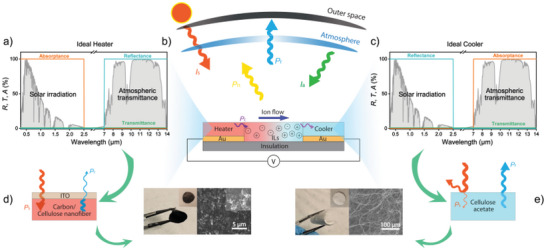
Concept of ionic thermoelectrics driven by solar heating and radiative cooling. a) Optical properties of an ideal heater, together with normalized spectra of solar irradiance and atmospheric transmittance in grey. b) Schematic illustration of light interaction and heat flows for a lateral ionic thermoelectric device driven by a radiative cooler and solar heater. c) Optical properties of an ideal cooler, together with normalized spectra of solar irradiance and atmospheric transmittance in grey. Schematic illustration of light interaction and thermal flows for a lateral ionic thermoelectric device driven by a cooler and heater. d) Schematic of the basic structure of the heater material together with photograph and scanning electron microscopy image of a final heater film. e) Schematic of the basic structure of the cooling material together with photograph and scanning electron microscopy image of a final sample.

**Figure** **1**b illustrates the overall device concept, with the cellulose heater and cooler placed on top of the electrodes of a lateral ionic thermoelectric device. The thermoelectric device generates an output voltage that linearly increases with the temperature difference between the two electrodes. Because the heater and cooler face the same environmental conditions in our lateral configuration, we rely on them having different visible and/or infrared optical properties in order to induce a temperature difference. In turn, maximized temperature difference is expected by maximizing the difference in net power of the thermal flows for the heater and the cooler, as both will follow the heat transfer equation:^[^ ^33^, ^34^ ^]^

(1) $$P_{\mathrm{net}}=P_{s}+P_{a}-P_{\mathrm{tr}}-P_{n}-P_{c}$$

where *P*_s_ is the absorbed power from incident solar radiation (*I*_s_), *P*_tr_ accounts for the power thermally radiated by respective materials, *P*_a_ corresponds to absorbed power due to incident atmospheric thermal radiation (*I*_a_), *P*_n_ is the thermal power lost or gained by non‐radiative heat exchange with the environment, and *P*_c_ corresponds to heat transferred through thermal conduction laterally within the device (due to temperature difference between the heater and cooler, Figure 1b). The arrows in Figure 1b indicate optical and heat exchange processes between the ambient and the system. If *P*_net, cooler_ ≠ *P*_net, heater_, the cooler and heater will find dynamic equilibria at different steady‐state temperatures at which their individual net heating/cooling power approaches zero, thereby generating a steady temperature gradient along the ionic thermoelectric electrolyte. Figure 1a,c illustrate the optical properties of an ideal heater and an ideal cooler, respectively. The ideal heater (Figure 1a) absorbs all visible and near‐infrared light to maximize *P*_s_ and reflects infrared light to minimize *P*_r_ lost through thermal radiation. The ideal cooler (Figure 1c) should instead reflect solar light to minimize *P*_s_ and absorb all infrared light to maximize *P_r_* and the corresponding cooling power.

We prepared our heater material by ultrasonic mixing of cellulose nanofiber solutions with carbon black, followed by drop‐casting. As shown in Figure 1d, this resulted in visibly black cellulose (BC) films, with thicknesses ≈50 µm. To lower the thermal emissivity in the MIR region and minimize its radiative cooling capability, the BC was coated by an MIR‐reflecting ITO layer to prevent thermal emission from the otherwise high‐emissive cellulose. As cooler material, we used electrospinning to prepare freestanding porous cellulose acetate (CA) films, with thicknesses ≈250 µm. The microporous structure results in broadband scattering in the visible region and makes the material appear white (Figure 1f). The cooler material did not comprise any ITO top layer because it needs the strong thermal emissivity of the cellulose to act as efficient radiative cooler. More details and illustrations of the preparation processes of the heater and cooler materials can be found in the Methods section and in Figure S1 (Supporting Information). The heater and cooler were cut into 1.2 cm × 1.2 cm squares for all measurements.

## 2 Results and Discussions

**Figure 2:**
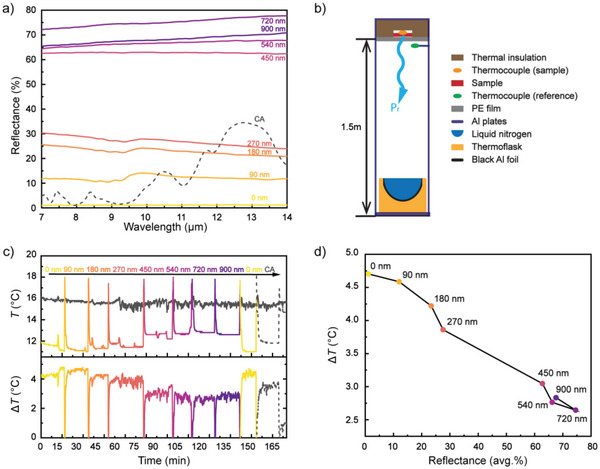
a) MIR reflectance of CA cooler and BC heaters with different thickness of ITO coating (from 0 to 900 nm). b) Schematic illustration of the sky simulator setup used to monitor radiative cooling performance of samples. c) Radiative cooling measurement using the sky simulator for CA cooler and BC heater materials with different thicknesses of ITO coating. d) Average Δ*T* extracted from (c) as a function of average reflectance in the wavelength range of 7–14 µm for the BC samples.

We begin the detailed characterization by investigating the MIR and cooling properties of cooler and heater materials. As mentioned above, cellulose‐based materials facilitate strong emission in the infrared because of molecular vibrations. This forms a natural advantage when cellulose is used for radiative cooling, but it is instead a drawback when optimizing a heater, for which the radiative cooling should be suppressed. ITO can provide high broadband transmittance of over 80% in the visible region and high reflectance in the infrared region.^[^ ^35^ ^]^ We therefore explored ITO as a thermal blocking layer for the BC heater material, with the aim to allow solar radiation to pass and be absorbed by the black cellulose while reflecting MIR and suppressing thermal radiation from the material. **Figure** **2**a presents the MIR reflectance of a noncoated BC film, BC films coated by ITO films of different thicknesses, and also of a CA film (the cooler material). As expected, the noncoated BC film showed very low MIR reflectance (≈1%) in the wavelength range of 7–14 µm (Figure 2a). Adding ITO coatings gradually increased the MIR reflectance with increasing ITO thicknesses, up to ≈75 % for 720 nm ITO coating. The sample with highest thickness of 900 nm showed somewhat decreased MIR reflectance, which was also accompanied by an increase in sheet resistance despite the thicker coating (Figure S2, Supporting Information). This indicates that the effect is due to less high‐quality ITO for the thickest film rather than thickness‐dependent optical effects. This was confirmed for another set of samples which also showed decreased MIR reflectance together with increased sheet resistance when the ITO layer reached a certain thickness. By contrast, the sheet resistance of the same thicknesses of ITO deposited on glass did not show this behavior but it instead decreased monotonically with increasing thickness (Figure S2, Supporting Information). The difference in results for the two types of substrates may be due to higher roughness of the cellulose substrate. The effect was furthermore pronounced when the thickness of the ITO increased, which led to poorer conductivity and lower IR reflectance. The porous structure of the CA cooler material, as intended to suppress solar absorption, led to higher MIR reflectance (grey dashed line) compared to the noncoated flat BC sample. However, the MIR reflectance of the CA sample was still considerably lower than for the optimized ITO‐coated cooling material. This suggests that the ITO‐coated BC heater should have low thermal emission and thereby suppressed cooling capability while the CA should provide high thermal emission and strong radiative cooling.

Infrared thermal camera imaging of the same batch of samples confirms that the ITO‐coated samples emit less thermal radiation when being at the same temperature, again with the 720 nm sample providing best suppression of the emission (Figure S3, Supporting Information). This can be concluded from that sample showing lowest apparent temperature as perceived by the thermal camera, despite all samples being at the same absolute temperature. Hence, we stress that the differences in apparent temperature correspond to the difference in the thermal emissivity of the different samples and not to differences in their absolute temperature. In turn, monitoring differences in absolute temperature for samples with different emissivity will require another method than thermal camera imaging, because it is sensitive to both emissivity and temperature. We therefore use thermocouples to monitor absolute temperatures in this study.

To verify the radiation blocking effect of the ITO layer, we performed radiative cooling experiments using a custom‐designed indoor sky simulator. Compared with outdoor measurements, the sky simulator provides stable reproducible nighttime conditions suitable for detailed comparison between different samples measured sequentially. Figure 2b shows a schematic diagram of the sky simulator and detailed information is provided in the Experimental Section. In brief, a black IR‐absorbing aluminum foil cooled by liquid nitrogen functions as the cold outer space by absorbing thermal radiation from the sample and only emitting minimal thermal radiation back to the sample thanks to its low temperature. The sample was fixed at the top of the setup on a thermal insulating foam and sealed by an IR‐transparent polyethylene film. The sides of the setup are made from IR‐reflecting aluminum plates to guide also off‐normal thermal radiation from the sample to the cold source at the bottom. Two thermocouples were placed at different positions as shown in the diagram to measure the sample temperature and ambient temperature inside the setup. Although the sky simulator does not provide identical conditions as the cloudless sky, results obtained using this indoor setup were in good agreement with those measured outdoor under cloudless weather at night (Figure S4, Supporting Information) and similar setups have been shown useful for detailed comparisons also in previous reports.^[^ ^36^ ^]^

Figure 2c presents radiative cooling results for the same samples as reported in Figure 2a, measured sequentially using the sky simulator. The top panel shows the temperature evolution first of the BC heaters with and without ITO coatings (colored solid lines) followed by the CA cooler (grey dashed line). ∆*T* in Figure 2c,d corresponds to the reduction in the temperature of the samples compared to the temperature of the ambient in the sky simulator. The temperature spikes correspond to changes of samples. The ambient reference temperature was measured using a separate thermocouple inside the setup (grey solid line) and it remained stable at ≈16 °C during the whole 170 min continuous experiment. The clear differences in temperature between the different samples can therefore safely be assigned to variations in their thermal radiation power. The cooler (CA) shows effective cooling performance of 4 °C below the reference temperature, which is slightly less than the BC film without ITO due to its higher reflectance. We summarize the performance of the heater materials in Figure 2d, which presents the averaged Δ*T* as a function of averaged MIR reflectance in the wavelength range of 7–14 µm. The graph shows an approximate linear relationship between the measured radiative cooling effect and the MIR reflectance. The noncoated BC film could be cooled by 4.7 °C, while the BC film coated by the 720 nm ITO showed cooling of only 2.6 °C, which demonstrates the effectiveness of our designed radiative cooling blocking layer.

**Figure 3:**
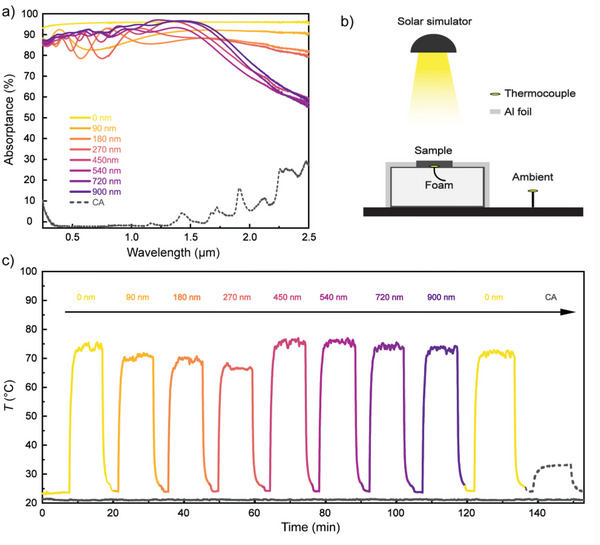
a) UV–vis‐near IR absorptance of a CA cooler (grey dashed line) and BC heaters with different thicknesses of ITO coating (colored solid lines). b) Schematic of the setup used to monitor the temperature of samples when exposed to the solar simulator. c) Solar heating measurement for CA cooler (grey dashed line) and BC heaters with different thicknesses of ITO coating (colored solid lines) using 1 SUN solar irradiance from the solar simulator (the illumination was turned off when samples were changed).

To investigate the solar heating performance of our materials, including possible effects of the ITO coatings, we first determined the UV–vis‐near IR absorptance of the heater and cooler materials using an integrating sphere (**Figure** **3**a). We first note that the CA cooling material and BC heater materials showed very different behavior. The CA cooler showed very low absorptance of only a few % throughout the visible range where the solar irradiance is strongest, followed by increase in absorptance toward the IR and for shorter wavelengths. These trends are consistent with previous works on porous cellulose materials.^[^ ^19^ ^]^ It is the high porosity of the CA cooler that minimizes the absorbed power from the sun, by reflecting light owing to its high density of optical scattering sites. By contrast, both the noncoated and ITO‐coated BC heater films instead showed high absorptance in the solar range, varying from around 80% to 95%. The oscillatory fluctuations in the spectra for the ITO‐coated BC films are due to optical interference effects and was indeed not present for the non‐coated BC. We note that the absorptance remained between 90 to 95% in the whole visible and near IR for the thickest ITO coatings. Those systems with thick ITO coatings are therefore promising for use as solar heater considering they also were the most efficient at suppressing thermal radiation in the mid‐IR. Indeed, suppressed emissivity can be discerned also in this figure from a decrease in absorptance at longer wavelengths for the BC heaters with thicker ITO coatings.

To more directly investigate the influence of the ITO layer and its thickness on solar heating, we monitored the temperature of the samples one by one while exposing them to light from a solar simulator as depicted in Figure 3b. Figure 3c shows the resulting temperature evolution of the BC heaters (colored solid lines), the CA cooler (grey dashed line) and the ambient (grey solid line) under 1 sun (AM 1.5, 1000 W m^−2^) solar irradiance. As expected, all BC films showed much higher solar heating (on the order of 50 °C) compared to the non‐absorbing CA film which only heated up by ≈11 °C. The solar heating among the BC films with different thickness of ITO did not show very large differences, which proves that the ITO coating does not hinder effective solar heating. Figure S5 (Supporting Information) shows the repeated solar heating results with error bars for three measurements. Notably, the 720 nm coating, which was found best at suppressing radiative cooling (Figure 2d), showed also among the highest heating performance, making it suitable as heater material for the following investigations.

**Figure 4:**
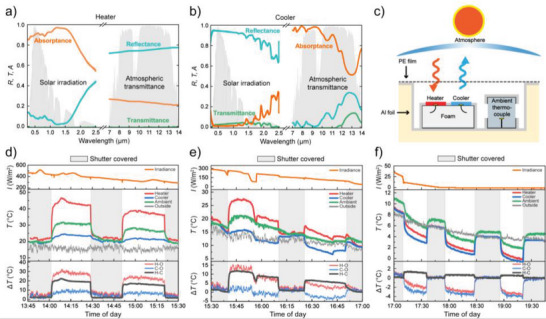
a,b) Optical properties of cooler (a) and optimized heater (b) in both the visible and MIR spectral regions. c) Schematic illustration of the outdoor setup used to monitor the temperature of a heater and a cooler when exposed to the sun and the sky. d–f) Continuous temperature measurement of cooler and optimized heater when exposed to the sun and the sky, separated into different panels for different solar irradiance ranges. The top part of each panel presents the measured solar irradiance. The middle part of each panel presents the temperatures and the bottom parts present differences in temperature between the heater and the outside (H—O), the cooler and the outside (C—O) and between the heater and the cooler (H—C). The shaded grey areas correspond to periods at which a shutter covered the setup.

Based on the combined results for radiative cooling and solar heating above, we choose the BC with 720 nm ITO as heater material and pure CA as cooling material for temperature measurements in real context outdoor. First, **Figure** **4**a,b presents the reflectance, transmittance and absorptance/emissivity of the optimized heater and cooler, which validate their suitable design and optical properties. The heater exhibited high solar absorptance of ≈90% in the range from 0.25–1.5 µm and also high MIR reflectance (70–80%) in the spectral range of the atmospheric window (7–14 µm), making it suitable for suppressing radiative cooling. The cooler shows qualitatively opposite behavior, with high reflectance of solar radiation and high absorptance in the MIR region, making it a natural thermal radiation emitter that is also good at suppressing solar heating (Figure 4b). Both heater and cooler further showed <5% transmittance in the whole UV–vis–NIR, effectively preventing influence from interaction of light with materials behind the films, such as the electrodes of our ionic thermoelectric devices as discussed below. To better understand how the optical differences between the cooler and heater may affect their thermal behavior (i.e., heat up or cool down) when exposed to the sun and the cloudless sky, we calculated their net radiative powers *P*_net,r_ = *P*_s_ + *P*_a_ − *P*_tr_ at different solar irradiances, as detailed in Supporting Information Note 1 and with results summarized in Table S1 (Supporting Information). For the cooler, we find that *P*_net,r_ is negative even at full solar irradiance (1 sun, ≈1000 W m^−2^, and with ambient temperature set to 20 °C). This indicates that the cooler should cool down and be able to provide sub‐ambient daytime radiative cooling. As expected, *P*_net,r_ for the heater is instead positive for all calculated solar irradiances (calculated in a range from ≈50–1000 W m^−2^), meaning that it will heat up and reach temperatures above that of the ambient. The exception is at zero solar irradiance (nighttime), for which also the heater provides negative *P*_net,r_ and should cool down instead of heating up.

We monitored the temperature of the heater and cooler simultaneously in the same measurement chamber so that they would be exposed to the same conditions (which are less stable over time outdoors) and to investigate the possibility to obtain temperature differences that can be used to generate thermovoltages in ionic thermoelectric devices. As illustrated in Figure 4c, the walls and floor of the setup chamber were covered with solar‐reflective aluminum foil to reflect incident light not hitting the samples and hence, to minimize absorption and heating by the setup itself. A polyethylene film, transparent in both the solar and MIR spectral ranges, covered the top of the setup to eliminate wind turbulence. We placed the heater and cooler on thermal insulation foam and monitored their temperatures using thermocouples placed under each sample. We monitored the temperature inside the measurement chamber using a thermocouple that was shaded from the sun and the sky using a small box with openings to allow for air circulation. We also used an additional thermocouple to monitor the outdoor temperature outside the measurement chamber. Figure 4d–f show the recorded temperatures, separated into different panels to highlight qualitatively different behavior during different times of the day due to variations in solar irradiance (as presented at the top of each panel). Shaded grey areas correspond to periods at which we covered the setup with a shutter. The bottom part of each panel shows three different temperature differences (Δ*T*): the temperature difference between the heater and the outside (H—O); the temperature difference between the cooler and the outside (C—O); and the temperature difference between the heater and the cooler (H—C, which will form the basis for inducing thermovoltages in thermoelectric devices). For the highest solar irradiances of ≈300–500 W m^−2^ (Figure 4d), the temperatures of the heater (red line), the cooler (blue line) and the ambient (green line) all showed an upward trend when exposed to the sun and the sky, although at different strengths. Although the absolute temperature slightly increased also for the cooler, we note that it remained lower than the ambient temperature in the measurement chamber, which is consistent with a calculated negative net radiative power as discussed above. An important outcome of the experiment is that the temperature difference between the heater and cooler (H—C) reached as high as 18–20 °C at these conditions. For intermediate solar irradiance within 75–300 W m^−2^ (Figure 4e), the cooler temperature started to show a downward trend when exposed to the sun and the sky, while the heater continued to show temperature increase. The turning point came at ≈225 W m^−2^ after which the cooler maintained temperature lower also than the outside temperature in the following part of the measurement. Hence, the heater and cooler showed opposite temperature trends at these conditions, leading to a clear temperature difference between the samples also at intermediate solar irradiances. For yet lower solar irradiances (Figure 4f), also the heater started to show a decrease in temperature when exposed to the sky, due to its nonzero cooling function and weakened solar heating. We note that the ambient temperature inside the box also gradually varied from being higher than the outside temperature at high irradiances to lower than the outside temperature at low irradiances. This is in agreement with the variation in the combined net radiative power from both samples, which changes sign at decreasing solar irradiances (Figure S6, Supporting Information). While both the heater and cooler showed temperature decrease at low and no solar irradiance, the decrease was always lower for the heater than for the cooler. This illustrates that the ITO‐coating of the heater indeed managed to act as a functional blocking layer for “trapping” the thermal energy inside the heater, leading to a temperature difference of ≈1 °C between the heater and the cooler even without solar irradiance. This highlights the possibility to use the materials for continuous operation of thermoelectric devices independent of day or night. Results from an overnight temperature measurement can be found in Figure S7 (Supporting Information).

**Figure 5:**
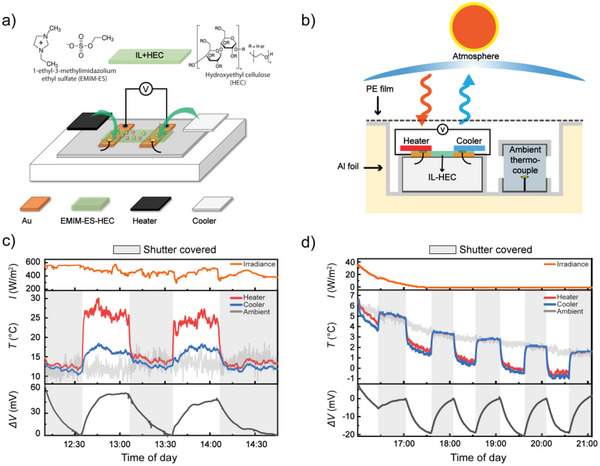
a) Schematic of the lateral thermoelectric device. b) Schematic of outdoor setup used to measure the generated thermovoltage powered by optimized heater and cooler when exposed to the sun and the sky. c,d) Continuous thermovoltage measurement of the lateral device when exposed to the sun and the sky, separated into two panels for daytime and nighttime. The top part of each panel presents the measured solar irradiance. The middle part of each panel presents the temperatures and the bottom parts present accumulation of open circuit thermovoltage. The shaded grey areas correspond to periods at which a shutter covered the setup.

After revealing the possibility to induce differences in temperature between the heater and cooler all day in outdoor conditions, we will demonstrate that these temperature differences can be used to power thermoelectric devices. **Figure** **5**a presents a detailed schematic illustration of the ionic thermoelectric device. Two gold electrodes with 1 cm separation distance were deposited on PET film which acts as a good thermal insulator owing to its low thermal conductivity. As ionic thermoelectric material, we used an electrolyte composed of the ionic liquid (IL) 1‐ethyl‐3‐methylimidazolium ethyl sulfate (EMIM‐ES) and hydroxyethyl cellulose (HEC) (Figure 5a). This gel‐like electrolyte provides good stability upon heating, high viscosity for the heater, and cooler adhesion, and potential for large thermovoltages thanks to the thermodiffusion of ions under a thermal gradient and dynamic water absorption/evaporation in the electrolyte.^[^ ^37^ ^]^ At ambient conditions (15 to 40 °C, 20%<RH<70%), the Seebeck coefficient (thermal voltage/temperature difference) of this electrolyte is between 6 to 13 mV K^−1^,^[^ ^37^ ^]^ which is much higher than that of electronic thermoelectric materials (see Figure S8, Supporting Information). Different from the vertical structure of thermoelectric devices that have been reported,^[^ ^28^, ^29^ ^]^ the lateral structure further allows water dynamics in the electrolyte films, which has been reported to contribute to the thermal voltage in addition to ionic thermodiffusion.^[^ ^37^ ^]^

We measured the thermovoltage of the thermoelectric device outdoors while simultaneously monitoring the temperature of the heater and the cooler, using the same measurement chamber as for the temperature outdoor temperature measurements above (Figure 5b). The measurement was carried out under a clear, cloudless sky, with a solar irradiance of 300–600 W m^−2^ in the daytime and close to 0 W m^−2^ at later times in the evening (Figure 5c,d). During daytime, the obtained temperature difference (Δ*T*) between the heater and cooler was ≈10 °C, which generated a clear thermovoltage across the ionic thermoelectric electrolyte (Δ*V*) of ≈66 mV. This corresponds to a Seebeck coefficient of 6.6 mV K^−1^. Although the energy harvesting principle of ionic thermoelectric materials is different from traditional thermoelectric generators, this would correspond to a figure of merit (*ZT*, based on charging efficiency) of around 0.02 and a power factor or 13 µW K^−1^m^−2^, based on the measured Seebeck coefficient, conductivity of around 3 mS cm^−1[^ ^37^ ^]^ and thermal conductivity^[^ ^38^ ^]^ of the material.

After sunset at close to zero solar irradiance, the device managed to maintain a clear stable temperature difference between the heater and cooler of ≈0.5 °C, thanks to the prevented cooling of the heater with ITO coating. When closing the shutter to the setup (grey shaded areas), both heater and cooler approached the temperature of the ambient. Repeatedly opening and closing the shutter also resulted in clear variations in the open‐circuit voltage of the ionic thermoelectric device. However, the change in voltage showed the opposite behavior from the daytime measurement, with the voltage increasing to positive values when exposing the device to the sky to start the cooling and induce the temperature difference. This phenomenon may be due to thermal voltages induced by the asymmetric device structure (electrodes covered by different materials),^[^ ^39^, ^40^ ^]^ which could then be the dominating effect compared to ionic thermodiffusion at small temperature differences.

## 3 Conclusion

In summary, we have presented an ionic thermoelectric device driven by radiative cooling, with and without the combination of solar heating. Under a cloudless sky with a solar irradiance of ≈400 W m^−2^, the device could obtain ≈10 °C temperature difference and generated ≈66 mV thermovoltage. This was achieved by designing two different cellulose‐based materials for use as solar heater and radiative cooler, respectively. Owing to opposite optical properties in the solar and MIR thermal spectral ranges, the heater and cooler materials could maintain clear and consistent temperature differences in both daytime and nighttime. Particularly the latter was enabled by suppressing thermal emission for the heater material using a MIR‐reflecting coating.

## 4 Experimental Section

### Heater Preparation

The solar absorber material was ITO deposited on black cellulose film prepared by casting process and magnetron sputtering (Vaksis 3 m, In_2_O_3_ 90 wt%, SnO_2_ 10 wt%). The schematic diagram of ITO/BC film fabrication process was shown in Figure S1 (Supporting Information). 0.1 g carbon black (CB) powder (acetylene, 50% compressed, 99.9+%, Thermo Scientific^TM^) was dispersed in 33.2 mL DI water by disperser (IKA® ULTRA‐TURRAX®) for 10 min. The mixture was transferred to ultrasonic water bath for 1 h at room temperature. 30 g CNF (0.1 wt%, 0.18 DS, 5 passes, RISE) and 0.03 g glycerol (99%, Sigma Aldrich) were added into the mixture and dispersed by disperser for 10 min. The mixture was transferred to ultrasonic water bath for 1 h at room temperature. 15 mL mixture was poured into petri dish (60×15 mm) and dried at 60 °C for 12 h to obtain the CB/CNF film. The ITO was deposited by magnetron sputtering under 62 W power and argon atmosphere (RF power) for a series deposition time to obtain ITO layer with different thickness.

### Cooler Preparation

The radiative cooler was cellulose acetate film with thickness of 250 nm prepared by electrospinning method (IME Technologies EC‐CLI). The schematic diagram of CA film fabrication process was shown in Figure S1 (Supporting Information). Detailed information about the process could be found in previous studies.^[^ ^19^ ^]^ Briefly, the 19 wt% cellulose acetate solution was obtained by using DMF and acetone (volume ratio 2:3) as co‐solvent under magnetic stirring. As shown in Figure S1 (Supporting Information), the cellulose acetate film was peeled off from the collector (aluminum foil) after electrospinning process (0.8 mm, 10 µL min^−1^, 25 kV). 15 mL CNF solution (0.06 wt%, 0.18 DS, 5 passes, RISE) was dried in petri dish (60×15 mm), the obtained cellulose acetate film was put on top of half‐dried gel‐like CNF and dried together at 60 °C for 2 h. The CNF layer was to protect the cellulose acetate film from being soaked by electrolyte.

### Characterization of Heater and Cooler

The microstructures of the samples were investigated by scanning electron microscopy (Sigma 500 Gemini, Zeiss AG). The thicknesses were measured by a surface profiler (Dektak 3^st^, Veeco) and screw digital micrometer. 5 places were randomly selected and measured, and the average value was taken as the thickness for each sample. The Reflectance, transmittance, and absorptance of heater and cooler materials were determined by spectral directional hemispherical reflectance (DHR) and directional hemispherical transmittance (DHT). The *R*, *T*, *A* in the UV‐vis‐NIR range(250–2500 nm) were measured by a spectrophotometer (Cary 5000, DRA‐2500 integrating sphere, *θ*_i_ = 8°) and detector (R928 PMT130 for UV and vis and cooled PbS for NIR). The *R*, *T*, *A* in the IR range(7–14 µm) were measured by Fourier Transform Infrared spectrometer (Bruker Vertex 70, Labsphere A562 integrating sphere, *θ*_i_ = 9°) and detector (DTGS).

### Indoor and Outdoor Temperature and Voltage Experiments

For indoor sky simulator, the inside of the thermoflask was covered with black IR‐absorbing aluminum foil (Rosco). The thermoflask was filled and cooled down by liquid nitrogen to simulate the cold outer space by absorbing thermal radiation from the sample. The whole set‐up was made of polished IR reflecting aluminum plates and guide the thermal radiation from the sample. There were two thermal insulating foam in the setup, one was used to fix the sample at the top of the setup, the other was under the thermoflask in order to insulate the thermal conduction of thermoflask and aluminum plates. Two thermocouples (K type, Pentronic AB) were placed at different positions measuring the sample temperature and ambient temperature inside the setup. For the indoor solar heating experiment, 1 sun irradiance was illuminated vertically on the samples using solar simulator (Solsim LCS‐100). The IR camera experiment was carried out by Flir Thermal Camera (ThermoVision A320G). For the outdoor measurement, we used a measurement chamber coated with aluminum tape. The cooler and heater were placed separately on two gold electrodes (90 nm) separated by 1 cm on a PET film (50 µm), as connected by a thin layer of ionic thermoelectric gel‐like IL‐HEC electrolyte. The electrolyte was applied evenly between the two electrodes. The heater and cooler were gently pressed against the electrolyte on each electrode. Two thermocouples (K type, Pentronic AB) were embedded in the electrolyte and covered by the heater and cooler to measure the temperature transfer from the heater/cooler to the electrolyte. The nanovoltmeter (2182A, Keithley) was used to measure the open voltage from the ionic thermoelectric device. ΔV was obtained as the change in the measured voltage using a linear baseline subtraction.

## Conflict of Interest

The authors declare no conflict of interest.

## Supporting information

Supporting Information

## Data Availability

The data that support the findings of this study are available from the corresponding author upon reasonable request.
