# Supplementary material for: Cellulose‐Based Radiative Cooling and Solar Heating Powers Ionic Thermoelectrics
Source: Adv Sci (Weinh). 2023 Jan 16;10(8):2206510. doi: 10.1002/advs.202206510 (PMC10015909; doi:10.1002/advs.202206510)
Supplement: Supplementary file 1 — Supporting Information [file ADVS-10-2206510-s001.pdf]

ADVANCED SCIENCE
Open Access

Supporting Information

for *Adv. Sci.*, DOI 10.1002/advs.202206510

Cellulose-Based Radiative Cooling and Solar Heating Powers Ionic Thermoelectrics

Mingna Liao, Debashree Banerjee, Tomas Hallberg, Christina Åkerlind, Md Mehebub Alam, Qilun Zhang, Hans Kariis, Dan Zhao and Magnus P. Jonsson**

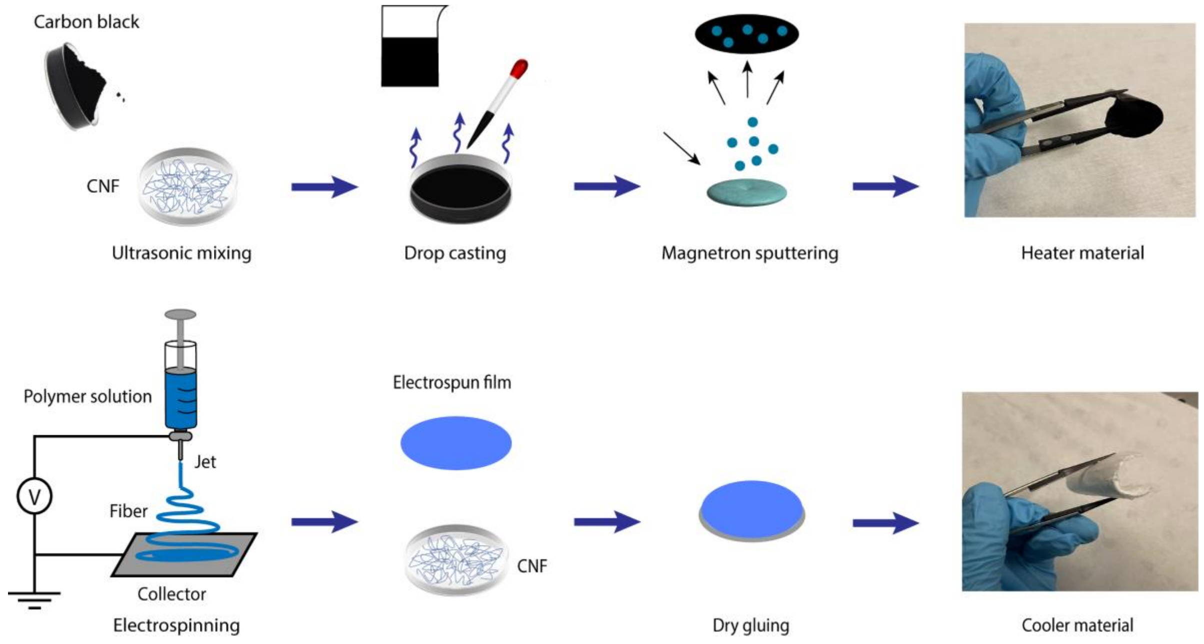

Figure S1. Schematic illustration of the fabrication process of heater and cooler materials.

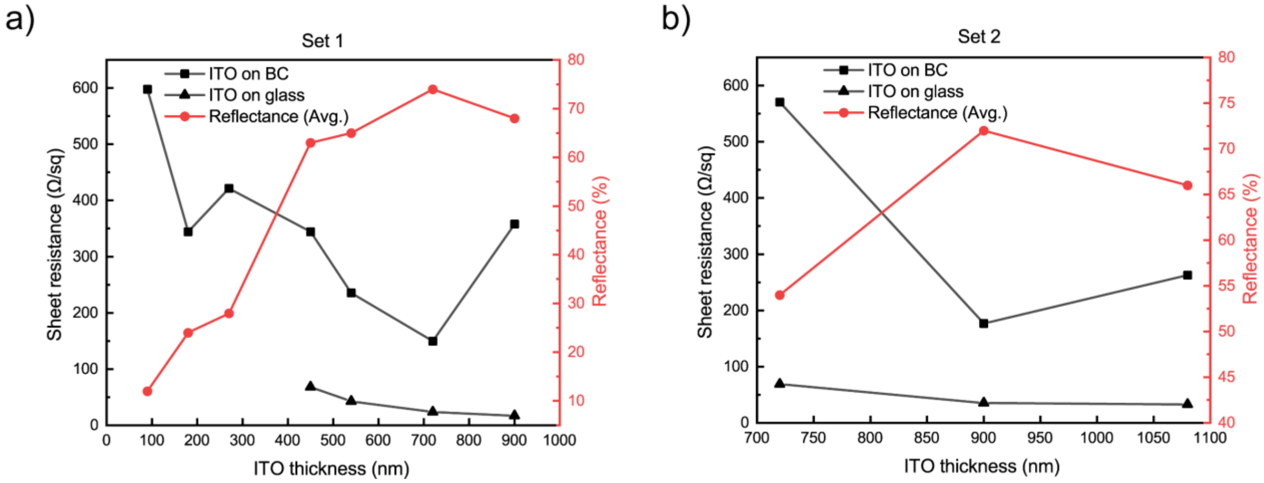

Figure S2. Average reflectance in the range of 7-14 µm MIR region (red, right axes) together with respective measured sheet resistance (black squares, left axes) for sample set 1 (a) and set 2 (b) of BC heaters with different thicknesses of ITO coating, and sheet resistance of ITO coatings on glass substrates (black triangles).

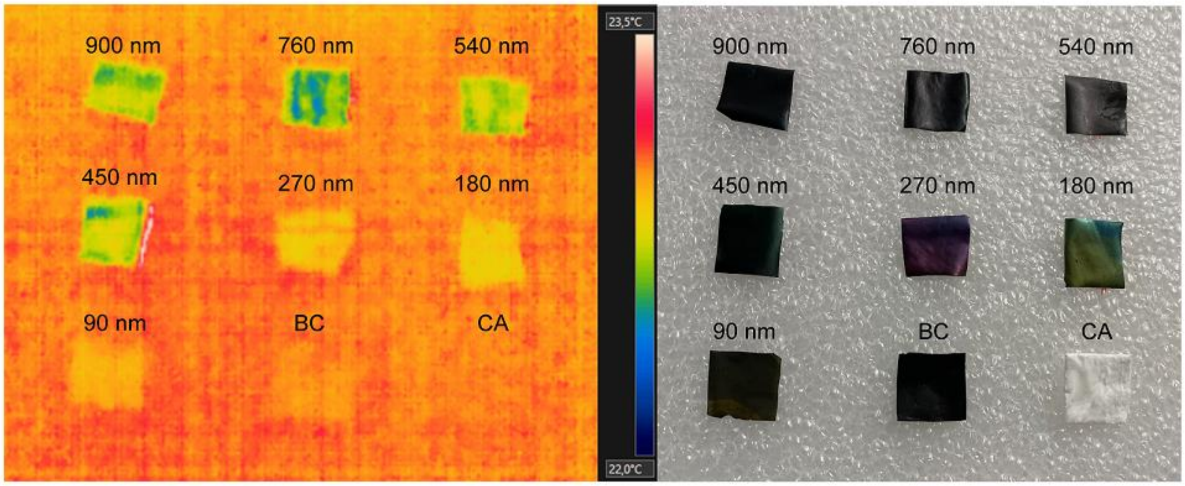

Figure S3. Thermal camera measurement results for CA cooler and BC heaters with different thicknesses of ITO coating at room temperature, obtained indoors using 0.95 emissivity as parameter.

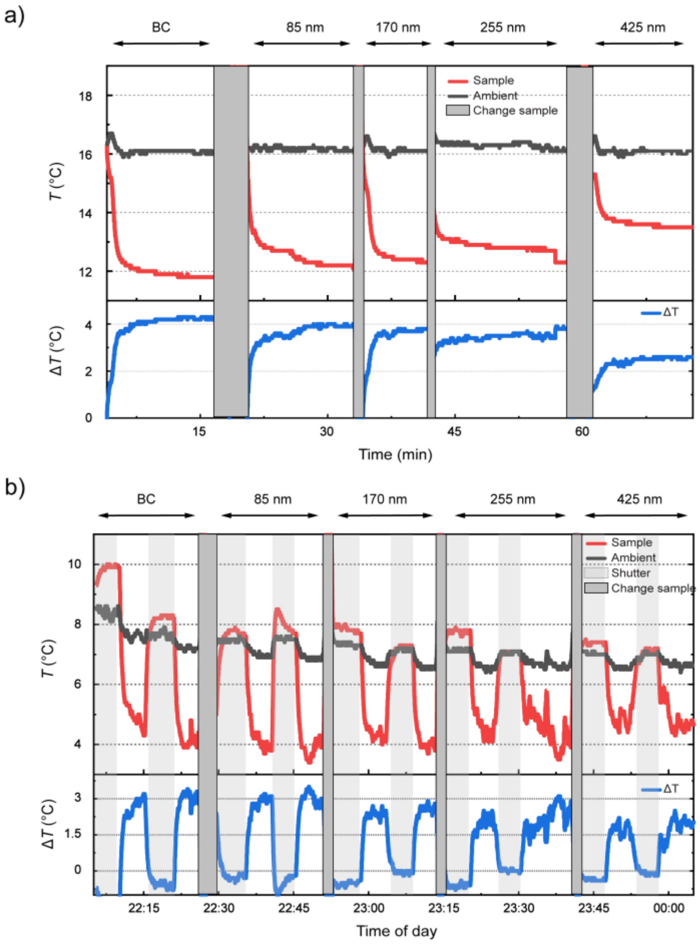

Figure S4. Temperature measurements of BC heaters (other sets) with different thicknesses of ITO coating in custom-designed indoor sky simulator (a) and outdoor environment (b).

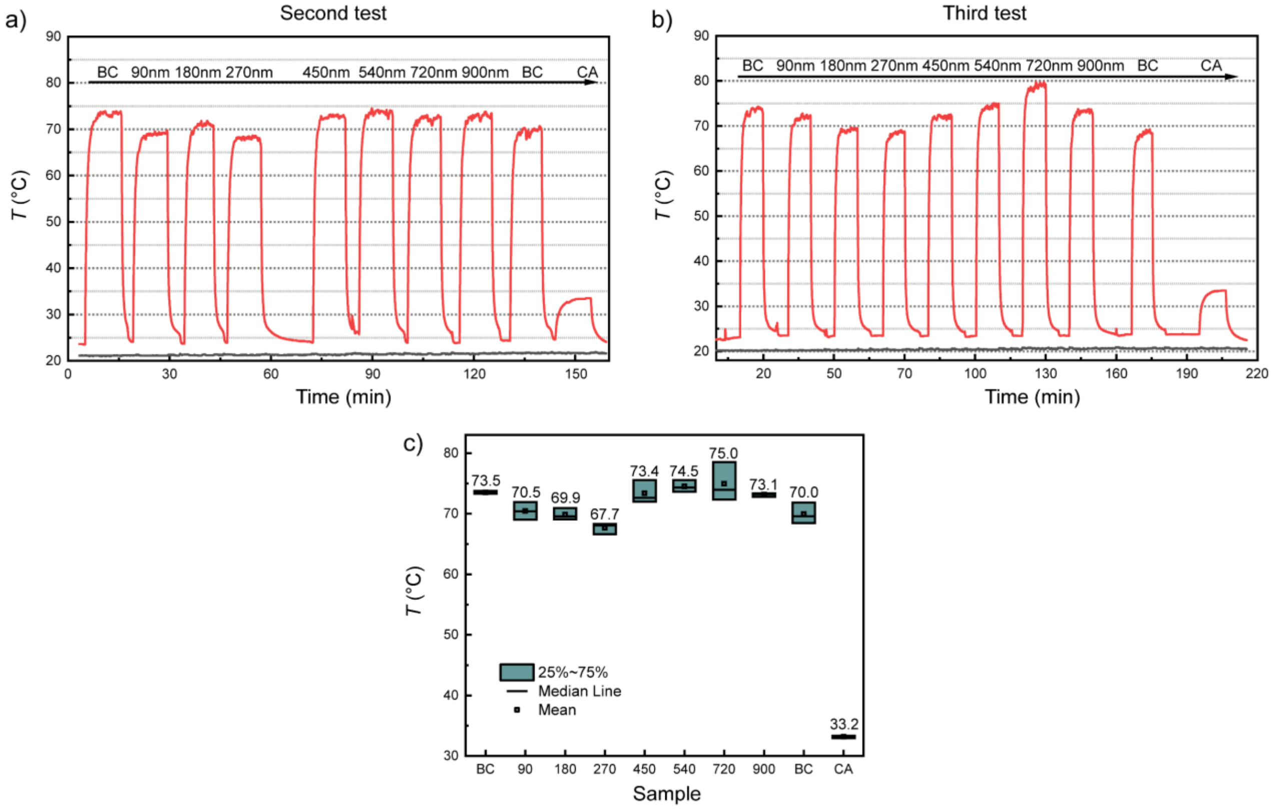

Figure S5. Repeated second (a) and third (b) solar heating measurements for CA cooler (grey line) and BC heaters with different thicknesses of ITO coating (red line) using 1 SUN solar irradiance from the solar simulator. (c) Summarized results including error bars from three solar heating measurements, with the mean values shown upon the boxes.

Supporting Information Note 1 - Calculations of net radiative powers for heater and cooler

The net radiative power for both the heater and cooler is given by:

$$P_{net,r} = P_{\mathrm{s}} + P_{\mathrm{a}} - P_{\mathrm{tr}}$$

where P_{s} is the absorbed power from incident solar radiation, P_{a} is the absorbed power from incident atmospheric thermal radiation, and P_{tr} corresponds to the thermal radiation from the sample. P_{s} can be calculated using:

$$P_{\mathrm{s}} = \int_0^{\infty} n I_{AM\ 1.5}(\lambda)\varepsilon_{\mathrm{sample}}(\lambda)\mathrm{d}\lambda$$

Here $I_{AM\ 1.5}$ is the AM 1.5 solar spectrum with irradiance of around 1000 W m^{-2}, $\varepsilon_{\mathrm{sample}}$ is the emissivity of the sample (assumed independent of angle and temperature) and n is used to modulate the solar intensity ($n = 1$ for 1 sun). We use AM1.5 Global tilt standard solar spectra data ASTM G-173-03 (International standard ISO 9845-1, 1992) from PVEducation (https://www.pveducation.org/pvcdrom/appendices/standard-solar-spectra). P_{a} can be calculated via:

$$P_{\mathrm{a}}(T) = \int_{\Omega} \cos(\theta) \int_0^{\infty} I_{BB}(\lambda, T)\varepsilon_{\mathrm{atm}}(\lambda, \theta)\varepsilon_{\mathrm{sample}}(\lambda)\mathrm{d}\lambda\mathrm{d}\Omega$$

where $I_{BB}(\lambda, T)$ is the thermal radiation from a black body at temperature T and $\varepsilon_{\mathrm{atm}}(\lambda, \theta)$ is the angle-dependent emissivity of the atmosphere, further approximated as:

$$\varepsilon_{\mathrm{atm}}(\lambda, \theta) = 1 - t_{\mathrm{atm}}(\lambda, 0)^{1/\cos(\theta)}$$

where t_{atm} is obtained from atmospheric transmission data from the Gemini Observatory of 2005 (https://webarchive.gemini.edu/20210519-sciops--instruments--mid-ir-resources--spectroscopic-calibrations/atmospheric-transmission-data.html) and approximating the temperature of the atmosphere to be the same as the ambient.

P_{tr} can be calculated by [see e.g. M.A. Zaman, International Journal of Thermal Sciences 144 (2019) 21–26]:

$$P_{\mathrm{tr}} = \pi \int_0^{\infty} I_{BB}(\lambda, T)\varepsilon_{\mathrm{sample}}(\lambda)\mathrm{d}\lambda$$

Below, we present the calculated results at fixed ambient temperature of 20 °C.

Table S1. Calculated net radiative powers for heater and cooler at different solar irradiance.

Solar Irradiance (W/m²)	Heater				Cooler			
	Ps	Pa	Ptr	Pnet	Ps	Pa	Ptr	Pnet
993	898			866	70			-38
898	812			781	63			-45
798	722			690	56			-52
699	632			601	49			-59
599	542			511	42			-66
496	449	13	44	417	35	44	153	-73
397	359			328	28			-80
298	269			238	21			-87
198	179			148	14			-94
99	90			58	7			-101
0	0			-31	0			-108

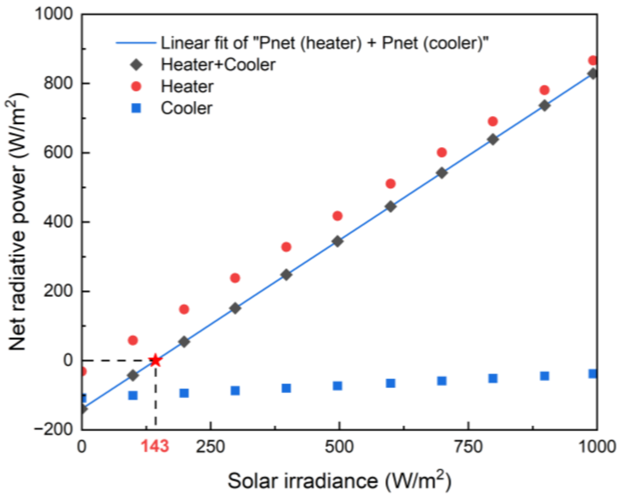

Figure S6. Calculated net radiative powers from Table S1 presented as a function of solar irradiance, also including the sum of the net radiative powers for the heater and cooler (i.e. assuming same areas).

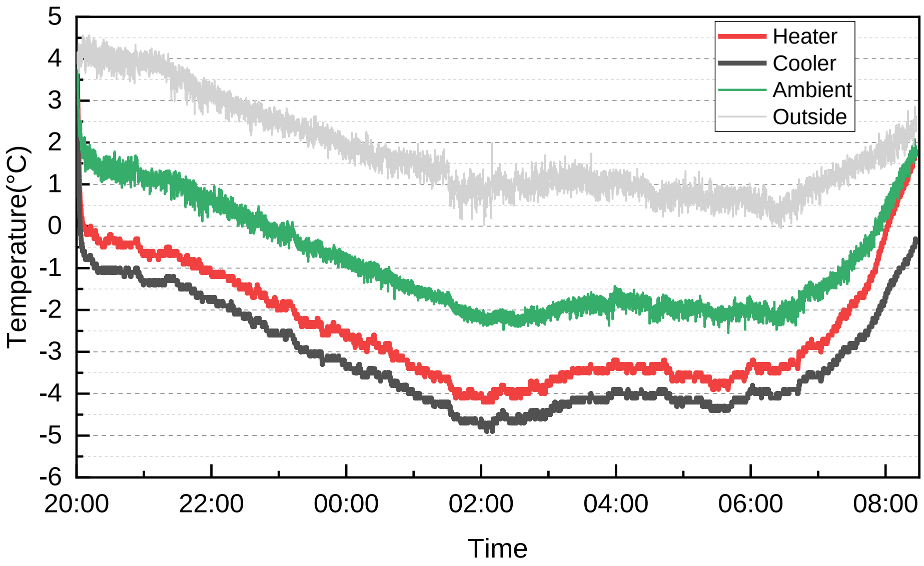

Figure S7. Overnight temperature measurement of a cooler and optimized heater exposed to the sky in outdoor environment.

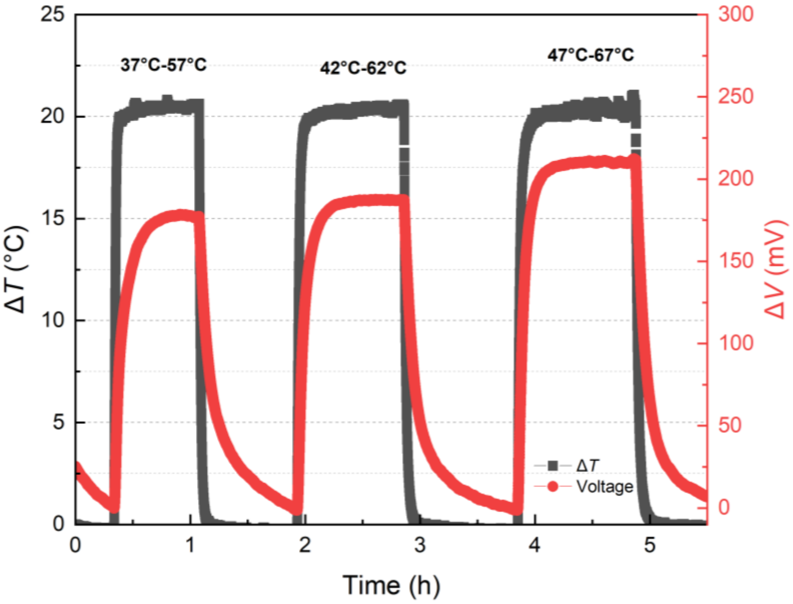

Figure S8. The generated current from the ionic thermoelectric device with stable temperature difference (ΔT=20K) under indoor condition.
